# Risk prediction for malignant intraductal papillary mucinous neoplasm of the pancreas: logistic regression versus machine learning

**DOI:** 10.1038/s41598-020-76974-7

**Published:** 2020-11-18

**Authors:** Jae Seung Kang, Chanhee Lee, Wookyeong Song, Wonho Choo, Seungyeoun Lee, Sungyoung Lee, Youngmin Han, Claudio Bassi, Roberto Salvia, Giovanni Marchegiani, Cristopher L. Wolfgang, Jin He, Alex B. Blair, Michael D. Kluger, Gloria H. Su, Song Cheol Kim, Ki-Byung Song, Masakazu Yamamoto, Ryota Higuchi, Takashi Hatori, Ching-Yao Yang, Hiroki Yamaue, Seiko Hirono, Sohei Satoi, Tsutomu Fujii, Satoshi Hirano, Wenhui Lou, Yasushi Hashimoto, Yasuhiro Shimizu, Marco Del Chiaro, Roberto Valente, Matthias Lohr, Dong Wook Choi, Seong Ho Choi, Jin Seok Heo, Fuyuhiko Motoi, Ippei Matsumoto, Woo Jung Lee, Chang Moo Kang, Yi-Ming Shyr, Shin-E. Wang, Ho-Seong Han, Yoo-Seok Yoon, Marc G. Besselink, Nadine C. M. van Huijgevoort, Masayuki Sho, Hiroaki Nagano, Sang Geol Kim, Goro Honda, Yinmo Yang, Hee Chul Yu, Jae Do Yang, Jun Chul Chung, Yuichi Nagakawa, Hyung Il Seo, Yoo Jin Choi, Yoonhyeong Byun, Hongbeom Kim, Wooil Kwon, Taesung Park, Jin-Young Jang

**Affiliations:** 1 Department of Surgery and Cancer Research Institute, Seoul National University College of Medicine, 101 Daehak-ro, Chongno-gu, Seoul, 03080 South Korea; 2 Department of Statistics and Interdisciplinary Program in Biostatistics, Seoul National University, 56-1 Shillim-Dong, Kwanak-Gu, Seoul, 151-747 South Korea; 3 Department of Mathematics and Statistics, Sejong University, Seoul, South Korea; 4 Center for Precision Medicine, Seoul National University Hospital, Seoul, South Korea; 5 Department of General and Pancreatic Surgery, The Pancreas Institute, University of Verona Hospital Trust, Verona, Italy; 6 Department of Surgery, Johns Hopkins University School of Medicine, Baltimore, USA; 7 Division of Gastrointestinal and Endocrine Surgery, Department of Surgery, College of Physicians and Surgeon, Columbia University, New York, USA; 8 Department of Pathology and Cell Biology, Columbia University Medical Center, New York, USA; 9 Department of Surgery, University of Ulsan College of Medicine, Asan Medical Center, Seoul, South Korea; 10 Department of Surgery, Institute of Gastroenterology, Tokyo Women’s Medical University, Tokyo, Japan; 11 Department of Surgery, International University of Health and Welfare Mita Hospital, Tokyo, Japan; 12 Department of Surgery, National Taiwan University Hospital and National Taiwan Hospital, Taipei, Taiwan; 13 Second Department of Surgery, School of Medicine, Wakayama Medical University, Wakayama, Japan; 14 Department of Surgery, Kansai Medical University, Osaka, Japan; 15 Department of Gastroenterological Surgery (Surgery II), Nagoya University Graduate School of Medicine, Nagoya, Japan; 16 Department of Surgery and Science, Faculty of Medicine, Academic Assembly, University of Toyama, Toyama, Japan; 17 Department of Gastroenterological Surgery II, Faculty of Medicine, Hokkaido University, Hokkaido, Japan; 18 Department of Pancreatic Surgery, Zhongshan Hospital, Fudan University, Shanghai, China; 19 Department of Surgery, Institute of Biomedical and Health Sciences, Hiroshima University, Hiroshima, Japan; 20 Department of Surgery, Hiroshima Memorial Hospital, Hiroshima, Japan; 21 Gastroenterological Surgery, Aichi Cancer Center Hospital, Aichi, Japan; 22 Pancreatic Surgery Unit, Division of Surgery, Department of Clinical Science, Intervention and Technology (CLINTEC), Karolinska Institute At Center for Digestive Diseases, Karolinska University Hospital, Stockholm, Sweden; 23 Department of Surgery, University of Colorado Anschutz Medical Campus, Denver, USA; 24 Department of Clinical Science, Intervention, and Technology (CLINTEC), Karolinska Institute, Stockholm, Sweden; 25 Department for Digestive Diseases, Karolinska University Hospital, Stockholm, Sweden; 26 Department of Surgery, Sungkyunkwan University School of Medicine, Seoul, South Korea; 27 Department of Surgery, Tohoku University, Tohoku, Japan; 28 Department of Surgery, Kobe University Graduate School of Medicine, Kobe, Japan; 29 Department of Surgery, Faculty of Medicine, Kindai University, Osaka, Japan; 30 Pancreaticobiliary Cancer Clinic, Yonsei University College of Medicine, Yonsei Cancer Center, Severance Hospital, Seoul, South Korea; 31 Department of Surgery, Taipei Veterans General Hospital and National Yang Ming University, Taipei, Taiwan; 32 Department of Surgery, Seoul National University Bundang Hospital, Seoul National University College of Medicine, Seoul, South Korea; 33 Department of Surgery, Cancer Center Amsterdam, Amsterdam UMC, University of Amsterdam, Amsterdam, The Netherlands; 34 Department of Gastroenterology and Hepatology, Amsterdam Gastroenterology Endocrinology Metabolism, Amsterdam UMC, University of Amsterdam, Amsterdam, The Netherlands; 35 Department of Surgery, Nara Medical University, Nara, Japan; 36 Department of Surgery, Osaka University Graduate School of Medicine, Osaka, Japan; 37 Gastroenterological, Breast and Endocrine Surgery, Yamaguchi University, Yamaguchi, Japan; 38 Department of Surgery, Kyungpook National University, Daegu, South Korea; 39 Department of Surgery, Tokyo Metropolitan Cancer and Infectious Diseases Center Komagome Hospital, Tokyo, Japan; 40 Department of General Surgery, Peking University First Hospital, Beijing, China; 41 Department of Surgery, Jeonbuk National University Medical School, Jeonju, South Korea; 42 Department of Surgery, Soonchunhyang University, Asan, South Korea; 43 Department of Gastrointestinal and Pediatric Surgery, Tokyo Medical University, Tokyo, Japan; 44 Department of Surgery, Pusan National University, Pusan, South Korea

**Keywords:** Gastroenterology, Oncology

## Abstract

Most models for predicting malignant pancreatic intraductal papillary mucinous neoplasms were developed based on logistic regression (LR) analysis. Our study aimed to develop risk prediction models using machine learning (ML) and LR techniques and compare their performances. This was a multinational, multi-institutional, retrospective study. Clinical variables including age, sex, main duct diameter, cyst size, mural nodule, and tumour location were factors considered for model development (MD). After the division into a MD set and a test set (2:1), the best ML and LR models were developed by training with the MD set using a tenfold cross validation. The test area under the receiver operating curves (AUCs) of the two models were calculated using an independent test set. A total of 3,708 patients were included. The stacked ensemble algorithm in the ML model and variable combinations containing all variables in the LR model were the most chosen during 200 repetitions. After 200 repetitions, the mean AUCs of the ML and LR models were comparable (0.725 vs. 0.725). The performances of the ML and LR models were comparable. The LR model was more practical than ML counterpart, because of its convenience in clinical use and simple interpretability.

## Introduction

Intraductal papillary mucinous neoplasms (IPMN) of the pancreas are premalignant lesions. The 2017 international consensus guidelines (ICG) on IPMNs proposed three high-risk stigmata and seven worrisome features as potential risk factors for malignant IPMNs^1^. Soon after, Kang et al. evaluated the hazard ratio (HR) of each risk factor listed in the ICG and demonstrated that the statistical significance differed among these factors because each risk factor had a different HR (3–9)^2^. Patients with IPMN routinely present with multiple different risk features of different degrees. Since then, models that can quantitatively predict malignancy have been deemed desirable.

Recently, several nomograms for quantitatively predicting malignant IPMNs were published^3–5^. The process of building these nomograms was mainly based on multivariate logistic regression (LR) analysis. These LR-based nomograms showed moderate prognostic predictability in the external validation with the area under the receiving operator curves (AUCs) ranging from 0.74 to 0.83.

Machine learning (ML) is a computational method that can establish ideal models for classification, prediction, and estimation by ‘automatically’ learning from a large-scale complex input and output dataset^6^. Recently, ML techniques have been utilized in a variety of medical fields, especially for diagnosing anticipated histopathology from radiologic images^7,8^, predicting disease prognosis^9^, and establishing models for differentiating benign and malignant diseases. For example, one study reported that a deep-learning-based model can detect early breast cancer from observed patterns of micro-calcifications in mammography with an accuracy of more than 85%^10^. Thus far, few studies have used ML techniques for predicting pancreatic malignancy. Therefore, the present study aimed to develop ML technique-based models for predicting malignant IPMNs using a multinational multi-institutional dataset and compare the diagnostic predictabilities of ML and LR techniques.

## Results

### Patient demographics and prognostic factors for malignant IPMNs in the multivariate LR analysis

A total of 3,708 patients, with a mean age of 65.4 years and a 1:4 male to female ratio, who had both clinical and radiological data were included in our study (see Table 1). This cohort included benign and malignant IPMN. The majority of pancreatic cysts in this cohort were located at the head (59.5%), followed by the body or tail (34.1%); 6.4% were diffuse type IPMNs with lesions in multiple locations. The mean cyst size was 30.3 mm, mean MPD diameter was 4.8 mm, and mural nodules were present in 1,285 patients (37.1%). In the multivariate LR analysis, age (OR 1.02, 95% CI 1.01–1.03, *P* < 0.001), sex (OR 1.22, 95% CI 1.05–1.42, *P* = 0.010), cyst size (OR 1.02, 95% CI 1.01–1.02, *P* < 0.001), MPD diameter (OR 1.24, 95% CI 1.20–1.28, *P* < 0.001), and presence of mural nodules (OR 2.38, 95% CI 2.05–2.78, *P* < 0.001) were independent risk factors for malignant IPMNs. Compared to the head lesions, body or tail lesions were significantly less malignant (OR 0.74, 95% CI 0.62–0.87, *P* < 0.001), and diffuse type lesions were more malignant (OR 1.54, 95% CI 1.14–2.08, *P* = 0.005).

**Table 1:** Predictive factors for malignant intraductal papillary mucinous neoplasm in the univariate and multivariate logistic regression analysis.

	Total (N = 3,463)	Univariate analysis			Multivariate analysis		
		Benign IPMN (N = 2094)	Malignant IPMN (N = 1369)	P value	Odds ratio	95% CI	P value
Age (mean ± SD, year)	65.4 ± 9.9	64.5 ± 9.8	66.7 ± 10.0	< 0.001	1.02	1.01 – 1.03	< 0.001
**Sex (No.)**				0.195			
Female	1,266 (36.6%)	784 (37.4%)	482 (35.2%)		Ref	Ref	
Male	2,197 (63.4%)	1,310 (62.6%)	887 (64.8%)		1.22	1.05 – 1.42	0.010
**Location (No.)**				< 0.001			
Head	2,059 (59.5%)	1,175 (56.1%)	884 (64.6%)		Ref	Ref	
Body or tail	1,180 (34.1%)	818 (39.1%)	362 (26.4%)		0.74	0.62 – 0.87	< 0.001
Diffuse	224 (6.4%)	101 (4.8%)	123 (9.0%)		1.54	1.14 – 2.08	0.005
Cyst Size (mean ± SD, mm)	30.3 ± 16.3	28.6 ± 14.5	33.6 ± 18.2	< 0.001	1.02	1.01 – 1.02	< 0.001
MPD diameter (mean ± SD, mm)	4.8 ± 2.5	4.2 ± 2.3	5.6 ± 2.5	< 0.001	1.24	1.20 – 1.28	< 0.001
Mural nodule (No.)	1,285 (37.1%)	576 (27.5%)	709 (51.8%)	< 0.001	2.38	2.05 – 2.78	< 0.001

IPMN, intraductal papillary mucinous neoplasm; MPD, main pancreatic duct.

### Selection of the best ML algorithm after tenfold CV

**Figure 1:**
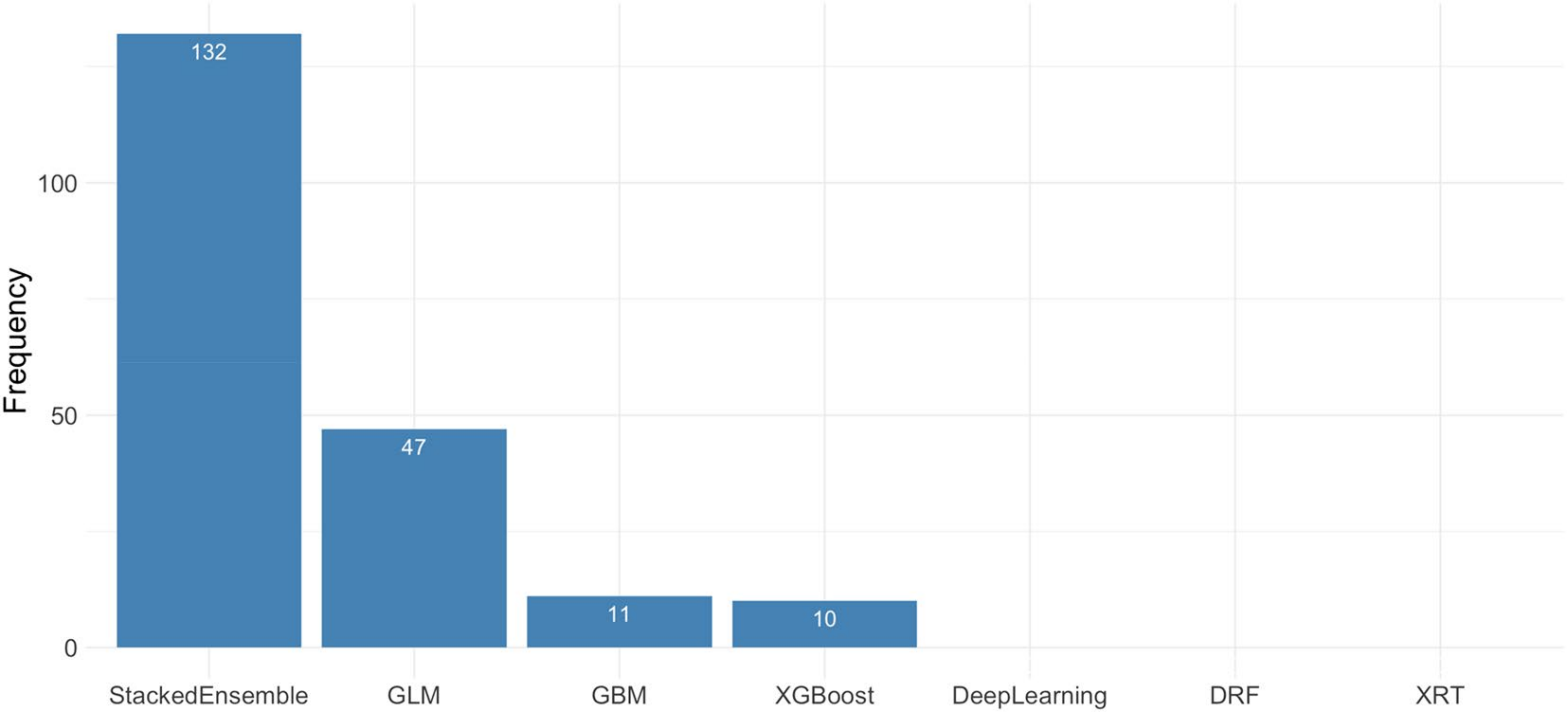
The number of the first ranked machine learning algorithm chosen in the tenfold cross validation during 200 times repetition.

**Figure 2:**
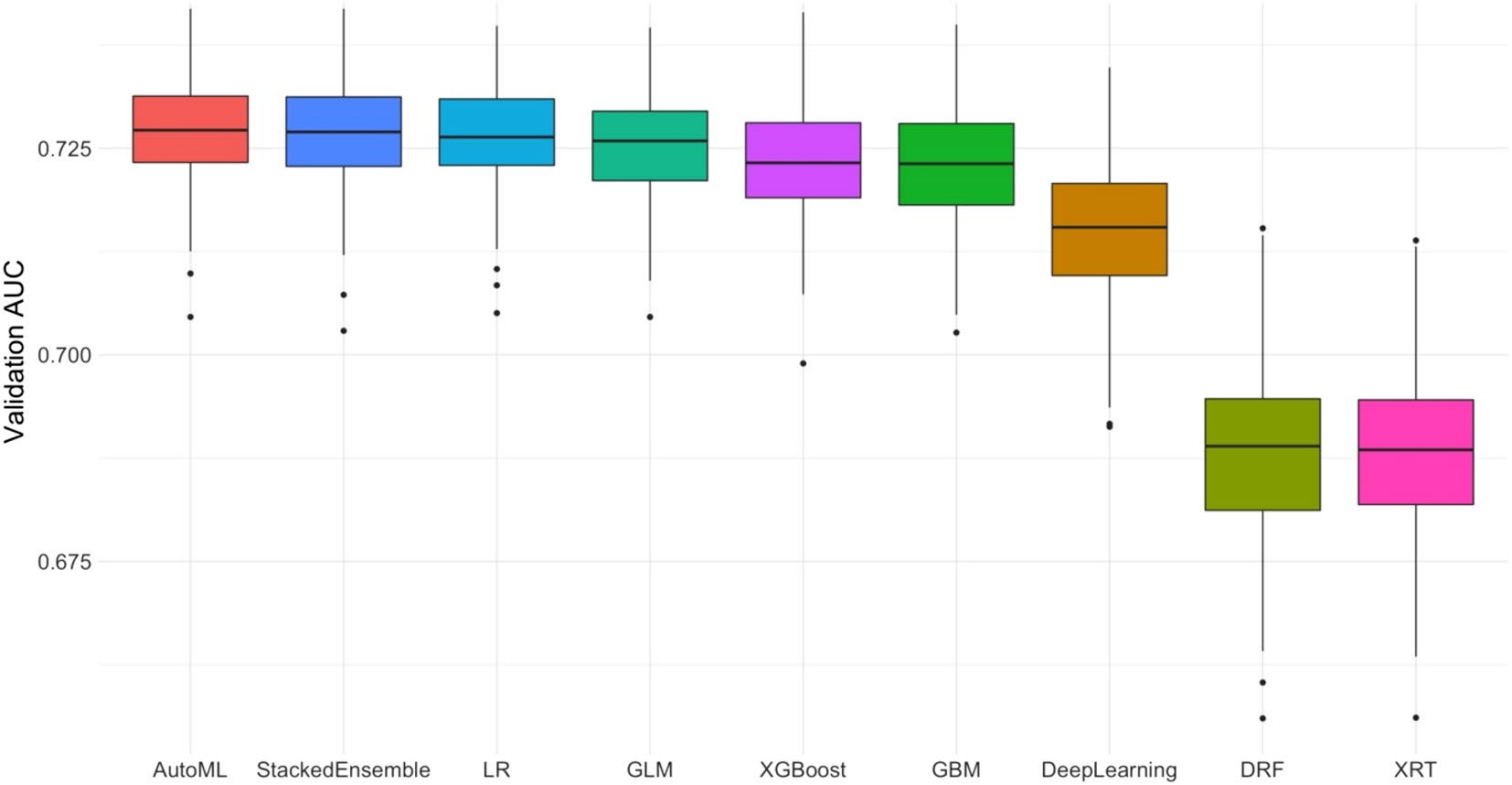
The mean highest tenfold cross validation are under the receiver operating curves of each algorithm during 200 times repetition. AUC indicates area under the receiver operative curve.

During 200 repetitions, we counted the number of ML algorithms that ranked first after the tenfold CV in each seed (see Fig. 1). SE was the most selected algorithm (n = 132), followed by GLM (n = 47), GBM (n = 11), and XG boost (n = 10). In addition, we calculated the highest tenfold CV AUC among each Auto ML algorithm in each random seed and evaluated the mean tenfold CV AUC for comparing the performance of each Auto ML algorithm. The SE algorithm had the highest mean AUC, followed by GLM, XG Boost, GBM, and DL (see Fig. 2).

### Comparison of the performances between ML and LR models

**Figure 3:**
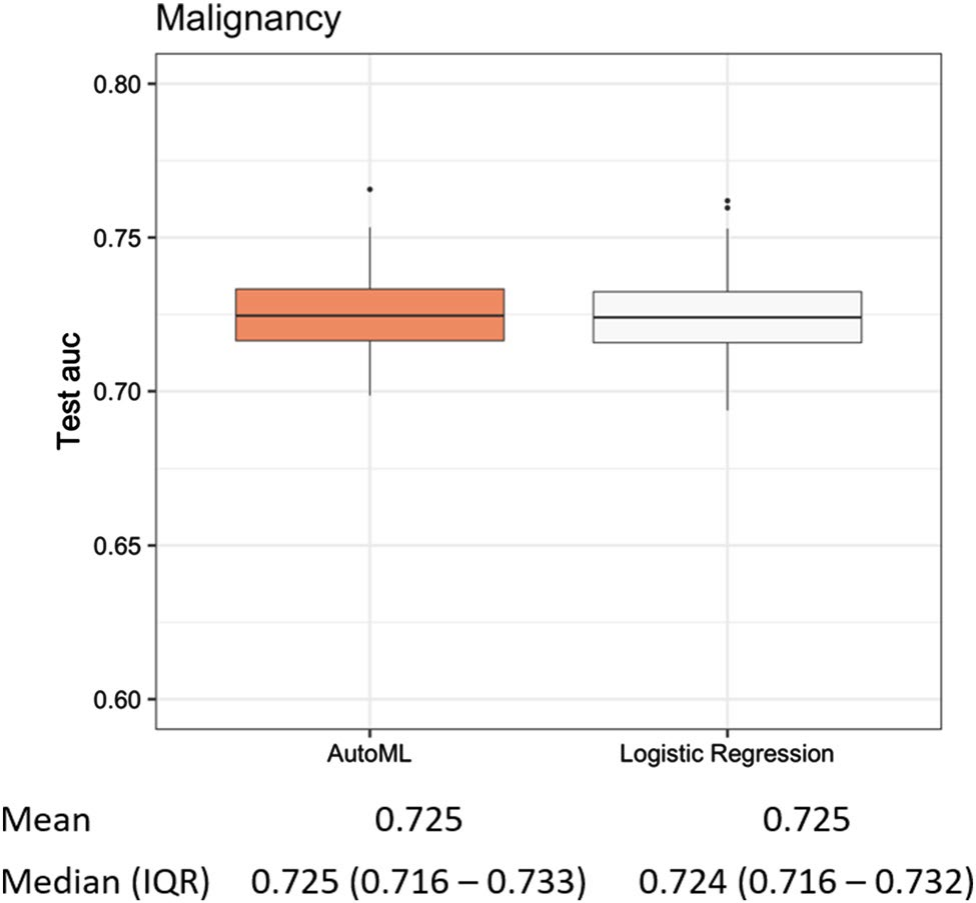
The overall performance of machine learning (ML) and logistic regression (LR). The performance of optimal ML model (Auto ML) was comparable with that of LR model (mean AUC, 0.725 vs. 0.725). AUC indicates area under the receiver operating curve.

Figure 3 shows the performances of AutoML and LR models after 200 repetitions. Overall, the mean AUC of both the models was 0.725.

## Discussion

**Figure 4:**
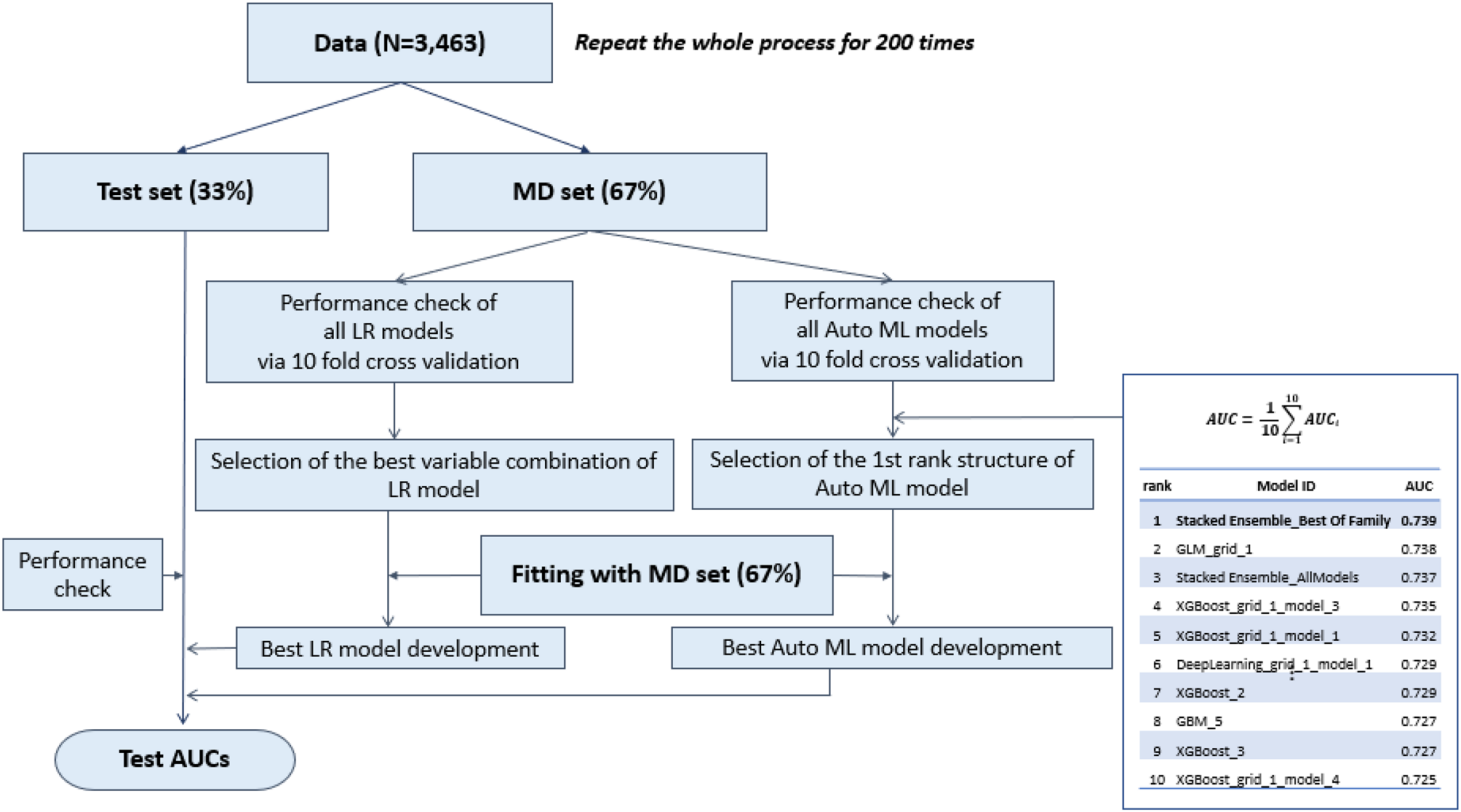
Overall flowchart of whole process. The workflows of both logistic regression (LR) and machine learning (ML) were separately processed in the same model development (MD) set. The whole process was repeated 200 times for reducing the selection bias which occurred during random split with test set and MD set. MD, model development; LR, logistic regression; Auto ML, automated machine learning; AUC, area under the receiver operating curve.

It has been established previously that each risk factor proposed in the 2017 ICG has different HRs^1,2^, hence models for predicting IPMN malignancy would need to be quantitative to accurately establish treatment strategies. LR has been widely used because of its simple structure and interpretability of coefficients. Several quantitative nomograms were developed with their own beta coefficient of risk factors based on the multivariate LR analysis^3–5^. For example, users can calculate and obtain the probability of malignant IPMNs easily and immediately, using a nomogram available at https://statgen.snu.ac.kr/software/nomogramIPMN. However, these nomograms showed similar moderate performances, in that, the AUCs did not exceed 0.85. In the current study, the LR model was established with several risk factors based on the multivariate LR analysis (see Table 1). To reduce the selection bias derived from random splits, these processes were repeated 200 times (see Fig. 4). The overall performance of the LR models was 0.725 (see Fig. 3), slightly lower than previous studies (0.72–0.85)^3,5,11^. To increase the performance, we hypothesized that prediction models based on different statistical techniques, such as the ML technique, can be potentially used as an alternative method for prediction and classification^12^.

ML algorithms have been utilised in a variety of medical applications in the twenty-first century. Due to faster data processing and improved computer functions, large number of data are processed in a short time leading to rapid advances in machine learning. ML algorithms can provide supportive information or additional aids for improving the accuracy and efficiency of diagnosis and treatment^13^, or aid in developing models to predict the prognosis^14^. The performance of models using ML algorithms is considered acceptable and comparable to human performance^15^. To evaluate the performance of ML in this study, LR was chosen as a baseline comparison.

The incidence of patients with pancreatic disease is quite rare; hence, it is difficult to apply ML algorithms for developing and validating the models in one institutional unit. Our study included over 3,708 patients from 31 institutions across 8 countries; therefore, the entire cohort consisted of a wide variety of ethnic groups across varied environments and health care systems.

**Figure 5:**
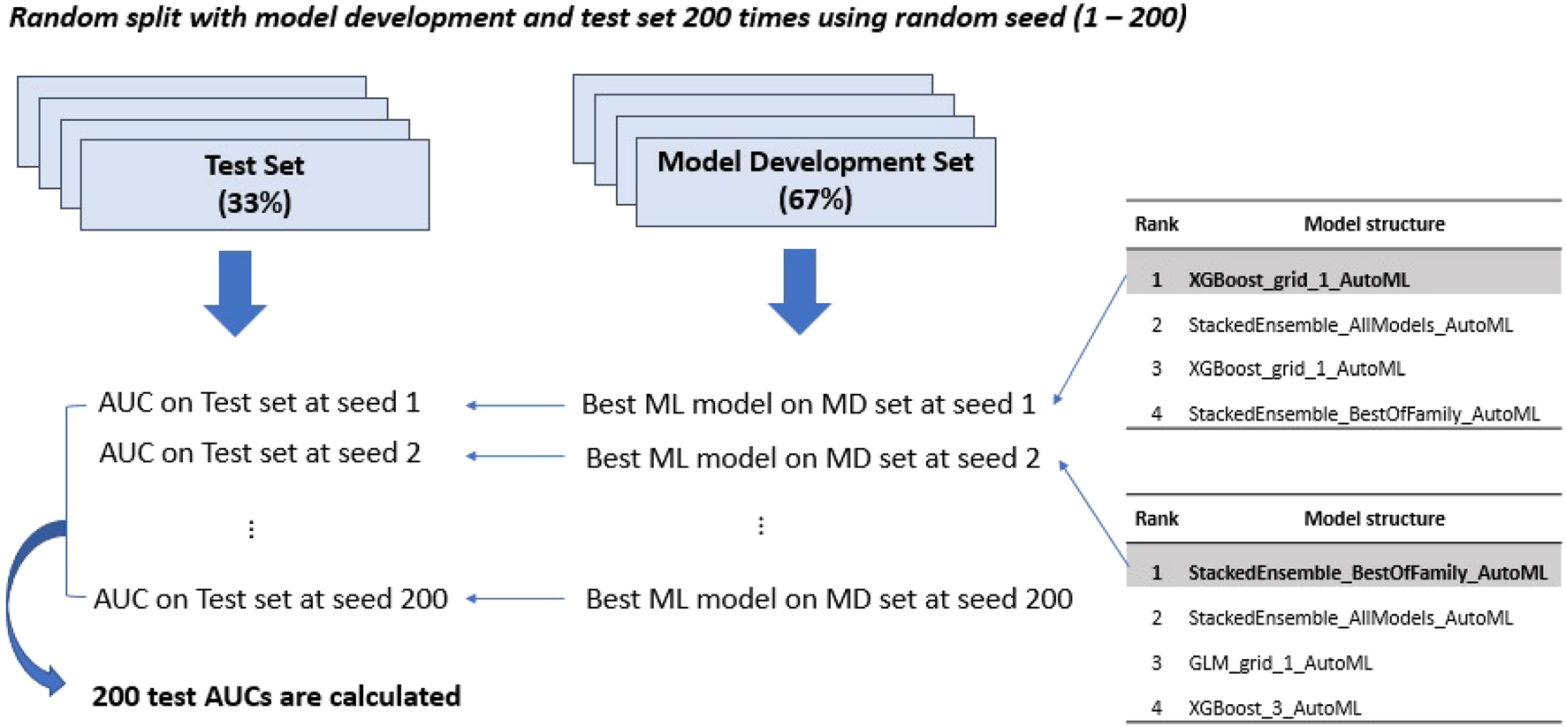
The process of calculation of test area under the receiver operating curves (AUCs) during 200 times repetition. After tenfold cross validation and selection of the first rank automated machine learning (Auto ML) model structure, this Auto ML model structure was fit with the model development set at each seed and the best ML model developed. Then the AUC was calculated with the test set. This process was repeated 200 times and mean AUC was calculated and compared.

Overfitting is one of the problems of a statistical model over-trained with the internal dataset, demonstrating unreliable performance and low diagnostic predictability when applied in the real world^16^. In our study, to overcome the overfitting problem and demonstrate real performance, the total dataset was divided into the MD and test set, and the model development and validation was performed on the two independent datasets (see Fig. 4). In addition, to reduce the selection bias during one random split, 200 repetitions were performed, and the mean test AUC was calculated (see Fig. 5); this reflected a reliable and accurate performance of ML and LR techniques in real practice.

The advantage of the ‘AutoML’ package program is that it automatically searches for the best ML algorithm and the best model for the particular structured data. After 200 repetitions, the mean test AUCs were comparable between the ML and LR models (0.725 vs. 0.725, see Fig. 3). In other words, both statistical techniques demonstrated the same performance in terms of developing models for the prediction of malignant IPMNs. Furthermore, we calculated the performance of each ML algorithm and counted the number of first-ranked ML model structures in each tenfold CV. Considering that the SE is an ensemble technique, the GLM had the highest mean tenfold CV AUC (see Fig. 1) among the independent AutoML algorithms, and it was selected more than the GBM, XG Boost, or DRF (see Fig. 2). In contrast with the GBM, XG Boost, and DRF, which were decision tree-based algorithms and fitted well with nonlinear association^17,18^, GLM and LR were based on linear regression analysis. These results indicated that the selected variables had a linear relationship with predicting malignant IPMNs, and the AutoML package program selected the algorithm that reflected the linear relationship as the best algorithm. If the variables with nonlinear relationships were involved in model development, the optimal ML algorithm might be changed.

Researchers developed ML models in a variety of medical fields and compared the performances of conventional LR and ML techniques. Some studies reported that ML models had more accurate predictability than LR models^19–22^, while others reported that ML and LR models had comparable predictability^23,24^. One study performed a systemic review and claimed that the performance of ML models was higher than that of LR models when ML models had a high risk of bias, and that the performances of ML and LR models were comparable when ML models had a low risk of bias^12^. Therefore, a more meticulous and accurate methodological approach is needed when conducting research using ML^12^. ML is not a replacement, but a complement, to LR. Therefore, the optimal statistical method can differ depending on the nature of the data or the purpose of the prediction problem.

Although the number of datasets were not sufficient to take advantage of ML, our study is the first to evaluate and compare the performances of ML models to LR in predicting pancreatic malignancy. The six variables had a relatively simple structure. Recently, ML techniques have been utilised to develop disease prediction models with high-dimensional omics data, such as the genomics and transcriptomics data, and these approaches outperformed existing prediction methods^25,26^. If the genomics or transcriptomics data on IPMN can be included in the future model development with ML techniques, the performance may be increased.

This study had some limitations. Because this study only enrolled the patients who underwent surgical resection due to IPMN, the results of this study did not represent the diagnostic performance in the general population in daily clinical practice. However, this study focused on the comparisons of diagnostic performance of two statistical methods, LR and ML. Although this was a retrospective cohort study with limited number of variables, the enrolled cohorts were multi-institutional and multinational. To prospectively enrol a large number of IPMN patients with standardised variables in a well-established collaborative study group would be desirable for future studies.

In summary, the performances of ML and LR models for predicting malignant IPMNs were comparable. The LR model would be more practical in clinical circumstances because of its simple interpretability and convenience in clinical use.

## Materials and methods

### Patients

The participating institutions in our retrospective cohort study with a multinational, multi-institutional medical database included 9 from Korea, 13 from Japan, 2 from China, 2 from Taiwan, 2 from the United States, 1 from the Netherlands, 1 from Sweden, and 1 from Italy. Patients who underwent a curative-intent surgical resection and had pathologic confirmation of IPMN between 1992 and 2017 were enrolled. Of all cohorts, patients who had both clinical characteristics (age and sex) and radiological characteristics (tumour location, cyst size, main pancreatic duct (MPD) diameter, and the presence of mural nodules) were included in our study. Tumour markers, such as carcinoembryonic antigen and carbohydrate antigen 19-9, were excluded during the analysis because they were not routinely evaluated preoperatively in the United States and Europe. According to the 2015 World Health Organization criteria, IPMN is graded as benign for a low-grade dysplasia and malignant for a high-grade dysplasia or an associated invasive carcinoma^27^. None of the cohorts had missing values.

Our study was approved by the institutional review board (IRB No. 1912-050-108) at Seoul National University Hospital, and the informed consents were obtained from all subjects. All methods were carried out in accordance with relevant guidelines and regulations.

### Preoperative radiologic evaluation

Preoperative radiologic parameters were evaluated with multi-detector computed tomography (CT) using either Brilliance 64 (Philips Medical Systems, Cleveland, OH, USA) or LightSpeed Ultra (GE Healthcare, Little Chalfont, UK), or magnetic resonance imaging (MRI) using Magnetom Verio (Siemens Healthcare, Erlangen, Germany). The tumour location was categorised as the head, body, tail, and diffuse. The cyst size, MPD diameter, and mural nodules were mainly measured from cross-sectional CT or MRI images and by using endoscopic ultrasonography (EUS) as required. All detectable mural nodules were recorded regardless of their size. Patients with MPD diameters greater than 10 mm in size were excluded from our study, as the definite main-duct type IPMN was not considered.

### ML model structure generation

We utilised ‘Automated machine learning (AutoML)’ in the H2O package from R program ver. 3.3.3 (R Foundation for Statistical Computing, Vienna, Austria) to automatically generate ML model structures based on seven ML algorithms: XG Boost, deep learning (DL), distributed random forest (DRF), generalised linear model (GLM), gradient boosting machine (GBM), extremely randomized trees, and stacked ensemble (SE). SE is an ensemble method that makes final predictions by incorporating decisions made from different models trained from other algorithms^28^.

For the attributes, for LR model we used logit link function and iteratively reweighted least squares (IWLS) estimation which is the default algorithm in glm() function in stats v3.6.2 package. Likewise, for ML model we used default options for automl() function in H2O v3.3.0 package.

### Development and evaluation of ML and LR models

The overall workflows are depicted in Fig. 4. To perform the model development and validation independently, the cohort was randomly divided into a model development (MD) set and a test set (2:1) in each random seed. For the LR model, we calculated the tenfold CV AUC for all possible LR models fitted with each variable set from all possible combinations. The one with the highest CV AUC was selected as the best variable combination.

For the ML model, the complete dataset of all collected variables was utilised because Auto ML applied many different ML algorithms to find the best model for the given training data. The tenfold CV was performed to evaluate the performance of all Auto ML model structures generated by the H2O package, and the one with the highest tenfold CV AUC was selected. A similar approach was used to predict an acute kidney injury after liver transplantation using clinical variables^22^.

Thereafter, the MD set was applied to both the LR and AutoML models to determine the best LR and AutoML model, respectively. Finally, the performances of these two models were evaluated with the test set to calculate their test AUCs.

To reduce selection bias, the entire process of the MD and test set division, the best LR and ML model selection, and test AUCs calculation was repeated 200 times. Figure 5 shows the process of calculation of the test AUCs during the whole random seed (1–200) with the ML model. Similar repetitions and calculations were performed with the LR model. To compare the overall performances of the LR and ML techniques, mean test AUCs were evaluated and compared.

### Statistical analysis

Categorical variables were compared using the chi-square test. Continuous variables were compared using the Student t-test. Variables with *P* < 0.05 in the univariate analysis were entered into a multivariate LR model to find significant predictors and estimate the odds ratios (ORs) for the corresponding predictors. Data was considered statistically significant when *P* < 0.05 in 2-tailed tests. All statistical analyses were performed using IBM SPSS Statistics ver. 22.0 (IBM Co., Armonk, NY, USA) and R program ver. 3.3.3.

## Data Availability

The datasets generated during the current study are not publicly available due to our institutional review board prohibits publication of patient’s personal medical records.
